# Supplementary figures and images for: Cockroach bacteriocytes migrate into the ovaries for vertical transmission of the bacterial endosymbiont Blattabacterium
Source: Zoological Lett. 2026 Jan 7;12:2. doi: 10.1186/s40851-025-00257-0 (PMC12870964; doi:10.1186/s40851-025-00257-0)

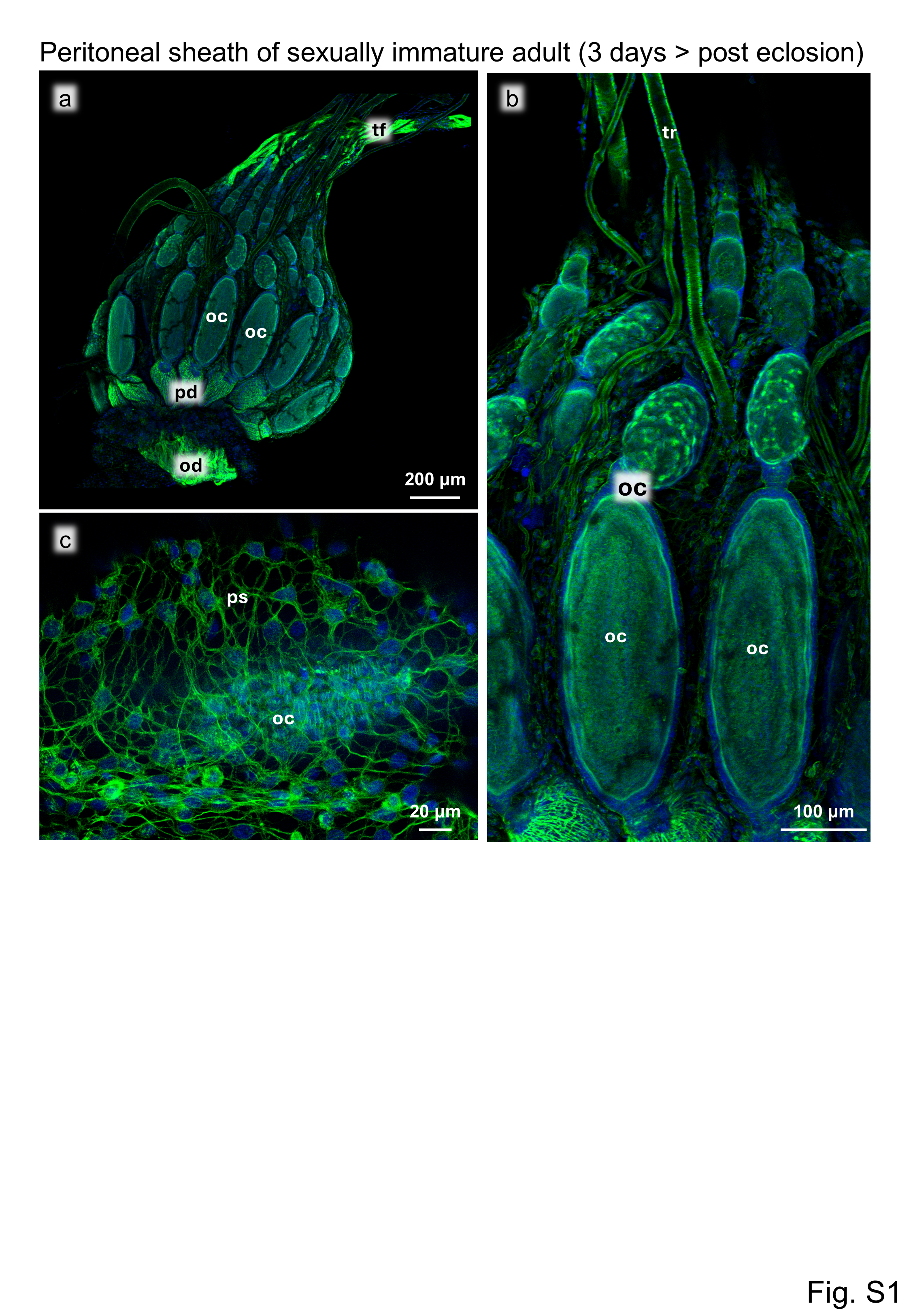

Supplement: Supplementary file 1 — Supplementary Material 1: Figure S1. Actin-stained ovary of a sexually immature adult. (a) Image of the whole ovary. (b) Magnified image of the ovarial surface. (c) Magnified image of the outer membrane (peritoneal sheath). Abbreviations: oc, oocyte; od, oviduct; pd, ovarial pedicle; ps, peritoneal sheath; tf, terminal filament; tr, trachea [file 40851_2025_257_MOESM1_ESM.tif]

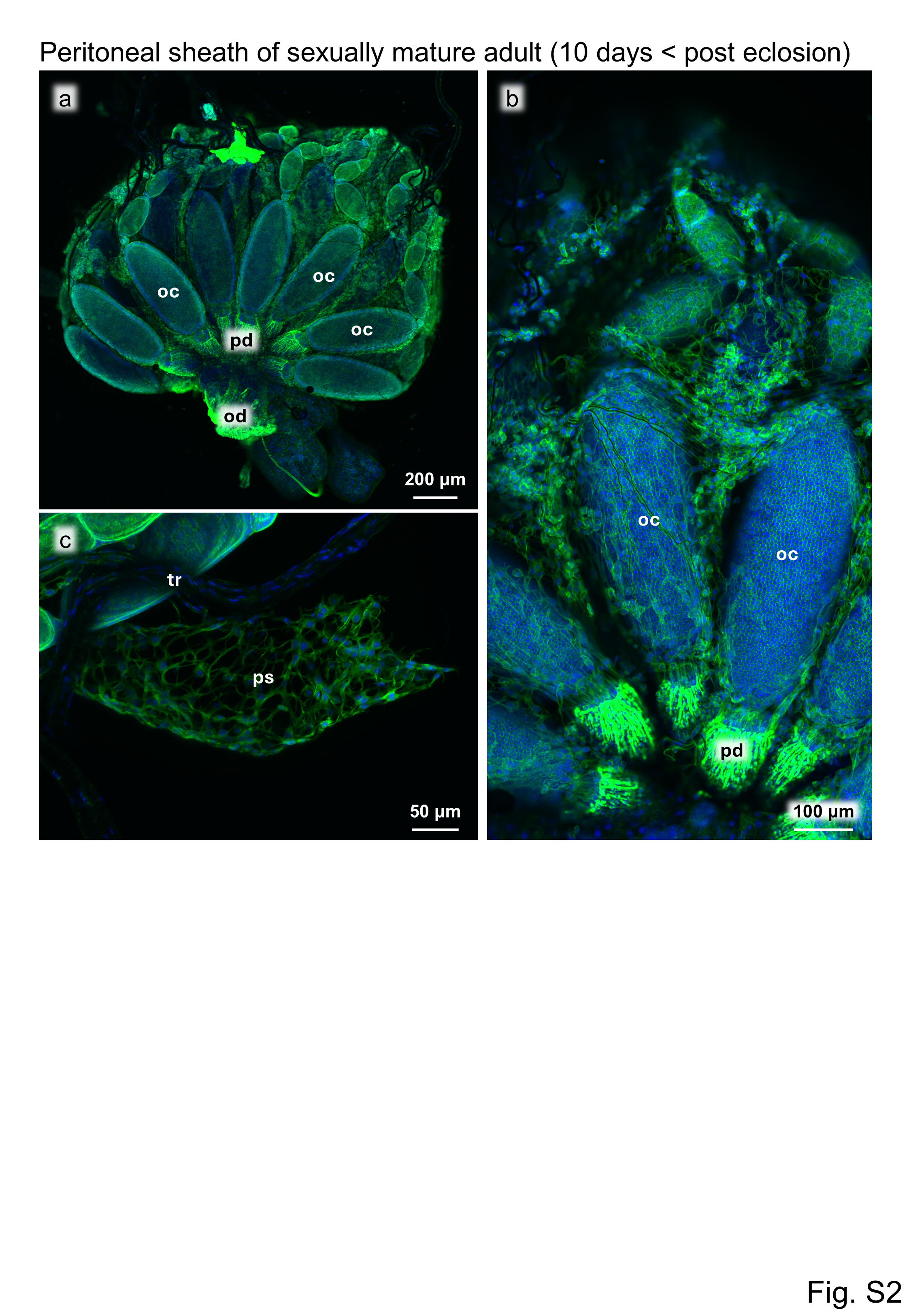

Supplement: Supplementary file 2 — Supplementary Material 2: Figure S2. Actin-stained ovary of a sexually mature adult. (a) Image of the whole ovary. (b) Magnified image of the ovarial surface. (c) Image of the partially removed outer membrane (peritoneal sheath). Abbreviations: oc, oocyte; od, oviduct; pd, ovarial pedicle; ps, peritoneal sheath; tr, trachea [file 40851_2025_257_MOESM2_ESM.tif]

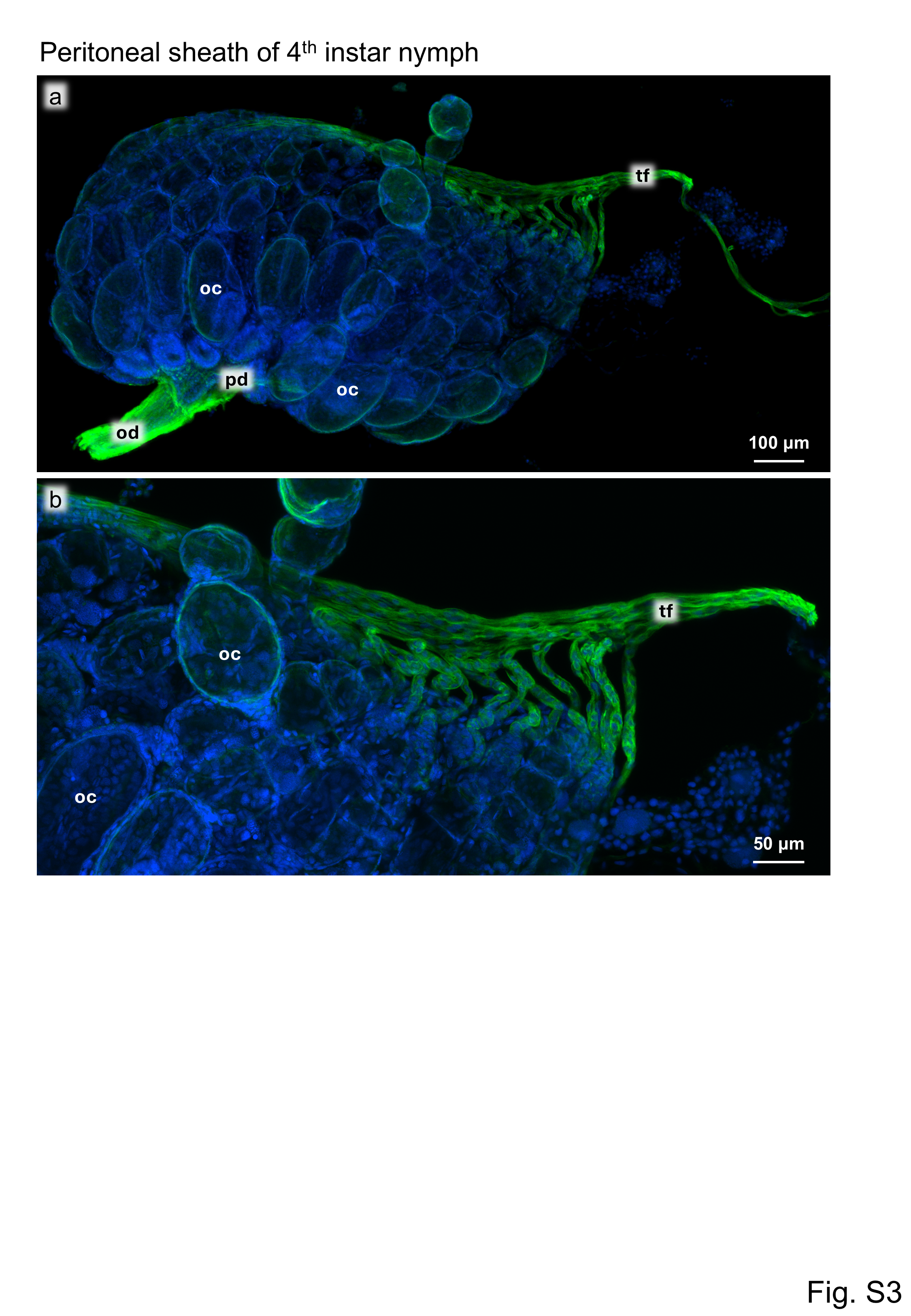

Supplement: Supplementary file 3 — Supplementary Material 3: Figure S3. Actin-stained ovary of a fourth-instar nymph. (a) Image of the whole ovary. (b) Magnified image of the ovarial surface. Abbreviations: oc, oocyte; od, oviduct; pd, ovarial pedicle; tf, terminal filament [file 40851_2025_257_MOESM3_ESM.tif]

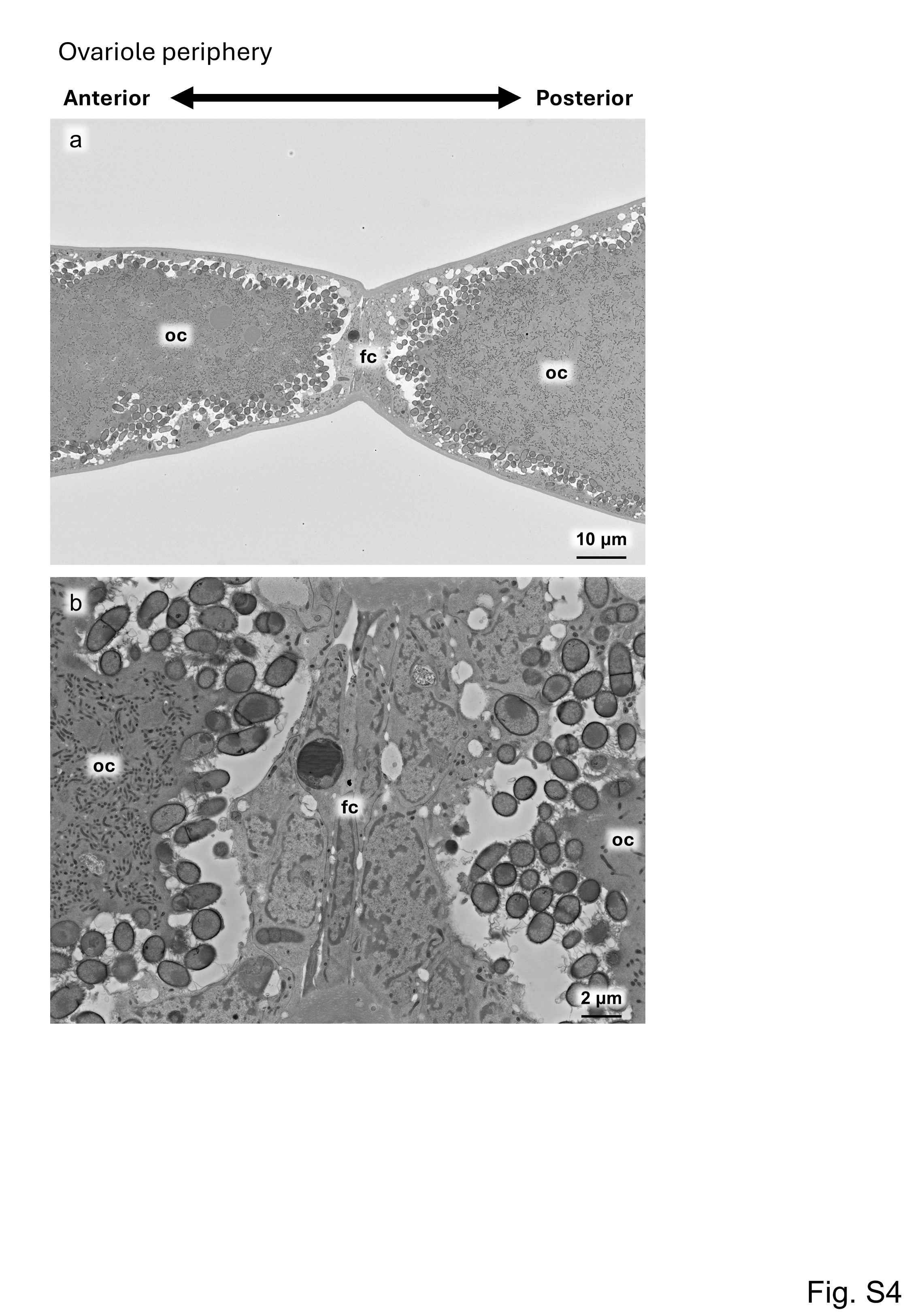

Supplement: Supplementary file 4 — Supplementary Material 4: Figure S4. Electron microscopic images of Zone IV of the ovariole periphery. (a) Wider view of the follicle cell layer separating the two oocytes. (b) Magnified image of the follicle cell layer. Abbreviations: fc, follicle cell; oc, oocyte [file 40851_2025_257_MOESM4_ESM.tif]
